# Sex differences in the peripheral levels of cytokines during 12-month antipsychotic treatment in a drug-naïve schizophrenia spectrum cohort

**DOI:** 10.1016/j.bbih.2025.100959

**Published:** 2025-02-03

**Authors:** I. Ratke, A. Torsvik, C.A. Bartz-Johannessen, F. Fathian, I. Joa, S.M. Klæbo Reitan, E.M. Løberg, M. Rettenbacher, S. Skrede, V.M. Steen, E. Johnsen, R.A. Kroken

**Affiliations:** a Haukeland University Hospital, Division of Psychiatry, Postboks, 1400, 5021, Bergen, Norway; b University of Bergen, Dr. Einar Martens Research Group for Biological Psychiatry, Department of Clinical Science 2, Postboks 7804, 5020, Bergen, Norway; c Haukeland University Hospital, Department of Medical Genetics, Postboks 1400, 5021, Bergen, Norway; d Stavanger University Hospital, TIPS – Network for Clinical Research in Psychosis, Postboks 8100, 4068, Stavanger, Norway; e University of Stavanger, Faculty of Health, Postboks 8600, 4036, Stavanger, Norway; f Norwegian University of Science and Technology, Department of Mental Health, Postboks 8900, 7491, Trondheim, Norway; g St. Olavs Hospital, Nidelv DPS, Department of Mental Health, Postboks 3250, 7006, Trondheim, Norway; h University of Bergen, Department of Clinical Psychology, Postboks 7807, 5020, Bergen, Norway; i Medical University Innsbruck, Department of Psychiatry, Psychotherapy and Psychosomatics, Anichstrasse 35, A-6020, Innsbruck, Austria; j Haukeland University Hospital, Department of Medical Biochemistry and Pharmacology, Postboks 1400, 5021, Bergen, Norway; k University of Bergen, Department of Clinical Medicine, Postboks 7804, 5020, Bergen, Norway; l Haukeland University Hospital, Mohn Research Centre for Psychotic Disorders (MRCP), Postboks 1400, 5021, Bergen, Norway

## Abstract

**Background:**

There are substantial sex differences in schizophrenia. However, research addressing sex differences regarding the antipsychotic effect on the immune system is lacking. The aim of our study was to compare changes in cytokine levels in men and women with schizophrenia spectrum disorder over 12 months of treatment with antipsychotics.

**Methods:**

This study reports pre-planned secondary outcomes from the BeSt InTro Study – a pragmatic, semi-randomised, rater-blinded comparison of amisulpride, aripiprazole, and olanzapine. The groups were analysed collectively. Of the 144 enrolled patients with schizophrenia spectrum disorders and ongoing psychosis, 56 were antipsychotic-naïve at baseline (20 women and 36 men) and were included in this study. Blood samples from these 56 patients were drawn at baseline, prior to treatment with antipsychotics, and 1, 3, 6, 12, 26, 39, and 52 weeks after initiation of antipsychotic medication. Duration of treatment was 52 weeks. Serum cytokine levels were assessed with a multiplex immunoassay. Changes in the levels of IL-4, IL-6, TNF-α, IL-1β, IL-2, IL-10, IL-12p70, IL-17A, IFN-γ and CRP from baseline to the different follow-up times were analysed using linear mixed effects models separately for men and women, and then compared.

**Outcomes:**

Cytokine levels were mainly stable in men during the study period. In women, IL-4 levels were lower at baseline compared with men (p = 0.048) and showed a consistent and significant increase at weeks 6 (p = 0.006), 26 (p < 0.001), 39 (p = 0.002), and 52 (p = 0.001). TNF-α increased in women at weeks 26 (p = 0.008) and 39 (p = 0.012). IL-6 had a transient increase in women at weeks 12 (p = 0.003) and 26 (p = 0.007). There were significant sex differences in progression of cytokine levels at weeks 3 (IL-6: p = 0.046), 6 (IL-4: p = 0.022, IL-6: p = 0.015), 12 (IL-6: p = 0.01), 26 (IL-4: p < 0.001, IL-6: p = 0.015, TNF-α: p = 0.026), 39 (IL-4: p = 0.003, TNF-α: p = 0.023) and 52 (IL-4: p < 0.001, TNF-α: p = 0.009). CRP levels did not differ between sexes at baseline or during the study period and did not change significantly during treatment with antipsychotics in either sex.

**Interpretation:**

We found significant sex differences in serum cytokine changes in drug-naïve patients with schizophrenia during treatment with antipsychotics. Cytokine levels were mainly altered in women, with increased IL-4, IL-6, and TNF-α levels. Cytokine changes may dramatically affect mental as well as somatic health. Our findings add to already established sex differences in schizophrenia pathophysiology and might have a potential role for future treatment guidelines.

**Funding:**

The Research Council of Norway, the Western Norway Regional Health Trust, and the participating hospitals and universities provided funding for this study.

## 1 Introduction

Schizophrenia is a severe and often chronic mental disorder frequently associated with a considerable disability and shortened life expectancy and with complex and multifactorial aetiology and pathogenesis (Hjorthoj et al., 2017). At a group level, a diagnosis of schizophrenia is associated with low-grade inflammation as measured by cytokines in the periphery (Upthegrove and Khandaker, 2020), cerebrospinal fluid (CSF) (Upthegrove and Khandaker, 2020; Wang and Miller, 2018), and brain (Foster et al., 2006; van et al., 2008). Altered immune activity has been associated with positive, negative, and cognitive symptoms of schizophrenia, as well as somatic comorbidity (Upthegrove and Khandaker, 2020; Fineberg and Ellman, 2013; Penninx and Lange, 2018).

Cytokines are proteins known to be involved in innate and adaptive immunity. They exert their effects in the periphery as well as the brain. As the evidence that immune system is involved in the pathophysiology of schizophrenia has accumulated, cytokines have been studied extensively in patients with schizophrenia, also regarding the impact of treatment with antipsychotics (Miller et al., 2011).

Antipsychotics affect immune activity at a cellular level and via various cytokines, but the effects on different cytokines are not fully understood. A 2011 meta-analysis covering cytokine levels in patients with first episode psychosis (FEP) who received antipsychotics showed a significant decrease in the plasma levels of several cytokines, namely interleukin 1β (IL-1β), IL-6, and interferon-γ (INF-γ) from Th1 cells, and IL-4 and IL-10 from Th2 cells. The IL-2 and IL-17 levels remained unchanged (Marcinowicz et al., 2021). Concerning non-FEP patients, the IL-1β, IL-6, and transforming growth factor-β (TGF-β) levels were reduced after treatment in patients with acute relapse, whereas the IL-12, IFN-γ, tumour necrosis factor-α (TNF-α), and soluble IL-2 receptor (sIL-2R) levels remained unchanged (Miller et al., 2011). In a 2018 meta-analysis on patients with schizophrenia, Romeo et al. reported a decrease in IL-1β and INF-γ and a trend towards a decrease in IL-6 and TNF-α following treatment with antipsychotics, whereas soluble TNF-R2 (sTNF-R2) and sIL-2R were increased (Romeo et al., 2018). Findings on how antipsychotics affect levels of C-reactive protein (CRP) are not consistent and differ between different drugs but there is a prevailing hypothesis of anti-inflammatory effects of antipsychotics (Fernandes et al., 2016; Lestra et al., 2022; Diaz et al., 2010; Fathian et al., 2022).

Interestingly, there is substantial sexual dimorphism in schizophrenia and immunity. In schizophrenia, there are sex differences regarding epidemiological distribution, comorbidity, psychopathology, morphological changes in the brain, and cognitive determinants (Fernando et al., 2020; Kelly et al., 2018). Oestrogens seem to play a positive role by reducing symptoms and improving cognitive performance (Brand et al., 2021a).

Sex as a biological variable also affects the pharmacokinetics and pharmacodynamics of antipsychotic drugs. Lower gastric acidity, slower gastric emptying, gastrointestinal transit and renal clearance, as well as higher body fat in women than men affects absorption, distribution and excretion of antipsychotics. This leads to higher plasma levels of antipsychotics in women, increasing risk for side-effects (Brand et al., 2021b). There have been reported sex differences in activity of several of the liver enzyme cytochrome P (CYP) like CYP1A, CYP2E1, CYP2D6 and CYP3A (Soldin et al., 2011). Preclinical evidence also indicates that oestrogens may influence on dopamine transporters (DAT) and increase sensitivity of D2 receptors in ventral tegmental area (VTA), leading to females achieving higher D2 occupancy compared to men at similar serum levels (Vandegrift et al., 2017). Women, at least perimenopausal, show better response to most of the antipsychotics, with the exception of amisulpride, risperidone and possibly clozapine (Brand et al., 2021b), but they also report higher burden of side-effects of antipsychotics than men (Iversen et al., 2018),with especially hyperprolactinemia being more pronounced in female patients (Bushe et al., 2008).

Sex dimorphism in immunity is found both in the innate and adaptive immune system, is prevalent across different species, not only humans, and is contributed to by sex chromosome genes, environmental exposure, microbiome, and sex hormones (Klein and Flanagan, 2016). Female sex is associated with altered humoral and cellular immune responses making them more susceptible for autoimmune diseases, while males are more prone to infectious diseases and non-reproductive malignancies. These immunological differences between sexes occur mainly during reproductive age (Klein and Flanagan, 2016).

Sex hormones have a profound effect on sexual dimorphism of the immune system and have been described to modulate differentiation, maturation, and the expression of innate immune cells (Klein and Flanagan, 2016). Oestradiol has a bipotential effect on immunity, with low doses elevating production of Th1 cytokines including IL-1β, IL-6, and TNF-α, whereas high concentrations have the opposite effect, with reduced production of IL-1β, IL-6, and TNF-α (Bouman et al., 2005). Oestrogen has also been shown to increase the levels of CRP (Wener et al., 2000). Progesterone, which is produced by the corpus luteum during the menstrual cycle and by the placenta during pregnancy, has a broad anti-inflammatory effect (Klein and Flanagan, 2016). Androgens have generally an immunosuppressive effect, reducing levels of TNF, and increasing levels of IL-1β, IL-2 and IL-10 (Klein and Flanagan, 2016).

Despite the numerous findings regarding sex differences in schizophrenia, immunity, and pharmacokinetics and pharmacodynamics of antipsychotics, there has been limited research regarding how antipsychotics affect the inflammatory status in women and men. Most studies correct for sex rather than stratify the results by sex. We aimed to fill this gap by examining how sex influences the fluctuation of inflammatory markers during antipsychotic treatment in patients with schizophrenia spectrum disorder. The objectives of this study were to measure cytokine levels in antipsychotic-naïve men and women before, during, and after 52 weeks of treatment with atypical antipsychotics (amisulpride, olanzapine, and aripiprazole), and to compare these levels between the sexes.

## 2 Methods

### 2.1 Ethical approval

This study was approved by the Regional Committees for Medical and Health Research Ethics and the Norwegian Medicines Agency in Norway (REK no. 2010–33876), and by the Etikkommission der Medizinische Universität Innsbruck and the Austrian Federal Office for Safety in Health Care (BASG) in Austria. This study has been registered on ClinicalTrials.gov (identifier NCT01446328).

### 2.2 Study design

This prospective, pre-planned secondary outcome cohort study was part of the BeSt InTro study – a pragmatic, randomised, rater-blinded comparison of amisulpride, aripiprazole, and olanzapine over 52 weeks. The outcome is part of the protocol approved by the ethical committees and competence authorities before the start of the trial. The participants were assessed at eight times – baseline and after 1, 3, 6, 12, 26, 39, and 52 weeks of treatment with antipsychotics. Baseline was defined as the day the participants started with study medication, and the first blood sample was drawn the same day, prior to intake of study medication. The primary outcome, the change in the Positive and Negative Syndrome Scale (PANSS (Kay et al., 1987)) score, has previously been published in *The Lancet Psychiatry* (Johnsen et al., 2020). In the present study, the results from all three treatment arms have been pooled, and the data have been analysed collectively through the 12-month follow-up. This study has been reported in accordance with the STROBE statement (von Elm et al., 2008).

### 2.3 Participants

The inclusion criteria for the BeSt InTro Study were: >18 years old; diagnosed with a schizophrenia spectrum disorder (F20–F29 in the International Statistical Classification of Diseases and Related Health Problems 10th Revision [ICD-10] from the World Health Organization); and ongoing psychotic symptoms with a minimum score of 4 on at least one of the following PANSS items: P1 (delusions), P3 (hallucinations), P5 (grandiosity), P6 (suspiciousness/persecution), or G9 (unusual thought content). The diagnoses were based on the Structured Clinical Interview for the Diagnostic and Statistical Manual of Mental Disorders IV axis 1 disorders (SCID-IV) (Kübler et al., 2013) with diagnoses converted to ICD-10. The participating patients met the indications for treatment with oral antipsychotics. The exclusion criteria were pregnancy or breastfeeding, prolactin (PRL)-dependent tumours, pheochromocytoma, hypersensitivity to the active substance or any excipients of the study drugs, concomitant use of levodopa or drugs that could induce torsade de pointes, known risk of narrow-angle glaucoma, and an inability to understand the native language.

The participants were recruited from three university psychiatric hospitals in Norway (Bergen, Stavanger, and Trondheim) and one in Austria (Innsbruck). Between October 20, 2011 and December 30, 2016, 359 patients were assessed for eligibility for the BeSt InTro study, and 215 of those were excluded (107 did not meet inclusion criteria, 82 declined to participate, and 26 for other reasons). The BeSt InTro study enrolled 144 patients, who were randomly assigned to receive amisulpride, aripiprazole, or olanzapine.

56 of these 144 patients in the BeSt InTro study were antipsychotic naïve and were therefore selected for this current analysis. Additional details concerning the BeSt InTro study are presented in a previous paper (Johnsen et al., 2020). Only samples from time points when patients were using medication were included in the analyses.

### 2.4 Medication

Fifteen patients received amisulpride (9 men and 6 women), twenty-three received aripiprazole (15 men and 8 women), and 18 received olanzapine (12 men and 6 women). The mean drug doses in the study were: amisulpride, 396.9 mg/day (standard deviation [SD] 206.9); aripiprazole, 14.6 mg/day (SD 7.0); and olanzapine, 12.3 mg/day (SD 3.8).

### 2.5 Procedures

At all study visits, blood samples were collected in the fasting state between 8 a.m. and 10 a.m. and left to clot at room temperature for 20–120 min, followed by centrifugation at 3300 rpm for 10 min. Serum was aliquoted, frozen at −40 °C, and transferred within 4 weeks for storage at −80 °C until analyses were performed.

The following cytokines were assessed with the High Sensitivity 9-Plex Human ProcartaPlex™ Panel (Thermo Fisher Scientific, Waltham, MA, USA): IL-1β, IL-2, IL-4, IL-6, IL-10, IL-12p70, IL17A, IFN-γ, and TNF-α. Analyses were run according to manufacturer's instructions, except for the universal assay buffer, which was replaced with 1X phosphate-buffered saline (PBS) with 0.10% Tween (PBS/Tween) to enhance fluorescence intensity. During the initial method validation, several analytes showed poor fluorescence intensities, likely due to matrix effects. Several buffers, additives, and dilutions were tested to improve the signals. The samples assayed in PBS/Tween showed acceptable values, and this buffer was used for all samples and standards. The samples were randomised with respect to sex and antipsychotic drug, but all samples from each individual participant were run on the same plate. The manufacturer's recommendations for sample dilutions and standard curve concentrations were followed, and all samples and standards were analysed in duplicate. The fluorescence intensities were measured on a Luminex 200 (R&D Systems, Inc., Minneapolis, MN, USA), with two lots of reagents covering all samples (21 plates). Both lots had comparable upper and lower limits of quantification (ULOQ/LLOQ), as specified in pg/ml: IFN-γ, 1193/1.16; TNF-α, 3960/0.97; IL-10, 810/0.20; IL-12p70, 4730/1.15; IL-17A, 760/0.19; IL-1β, 875/0.21; IL-2, 2570/0.63; IL-4, 3940/0.96; and IL-6, 763/0.74.

The standard curve was expanded from seven to eight dilutions to register more measurements around the LLOQ. The standard curve was added to all plates to avoid effect of plate-to-plate variance. Out of range (OOR) values (below the LLOQ) were set to LLOQ/2 to aid in calculations, in accordance with the method used by another group (Pfister et al., 2020).

CRP and prolactin were assessed by the regular hospital laboratories in Bergen, Stavanger, Trondheim, and Innsbruck (Hoprekstad et al., 2023).

### 2.6 Statistical analyses

The IFN-γ, IL-1β, IL-10, IL-12p70, IL-17A, IL-2, IL-4, IL-6, and TNF-α levels, as well as CRP, at baseline and changes during the study period were analysed with linear mixed-effects models. Due to skewness in the cytokine values, log-transformed values were used in the models. Age, body mass index (BMI), sex, time (weeks from baseline), and the interaction between sex and time were included as fixed effects, and patient ID was included as a random effect to account for dependencies in the data due to repeated measurements from the same individuals. The changes in cytokine levels from baseline to the different follow-up times were analysed for men and women, and cytokine levels at the different follow-up times were compared. Because of the explorative nature of the analyses, there was no correction for multiple comparisons. Because some of the analysed cytokines have a predominantly Th1 effect (Kroken et al., 2018; Pandurangi and Buckley, 2020) a combined standardised Th1 variable was constructed from the standardised means of IL-1β, IL-6, and TNF-α (Holdsworth and Gan, 2015).

### 2.7 Role of the funding source

The funder had no role in the study design, collection, analysis, or interpretation of the data; writing of the paper; or the final decision to submit the paper for publication.

## 3 Results

### 3.1 Demographics

At baseline, there were 20 women and 36 men in our sample (Table 1). The women were significantly older than men, with a mean age of 36.4 years (SD 15.1) compared to 28.3 years (SD 9.6) in men. More females (50%) than males (19.4%) received benzodiazepines at some point during the study. None of the women used oestrogen or progesterone medication. None of the participants used prednisolone. There was attrition during the study, and at week 52, three women and 9 men remained. See Table 4 for attrition week by week.

**Table 1:** **Demographic and baseline clinical variables by sex** BMI = body mass index. CGI=Clinical Global Impression. ICD-10 = International Statistical Classification of Diseases and Related Health Problems 10th Revision. NSAIDs = non-steroidal anti-inflammatory drugs. PANSS=Positive and Negative Syndrome Scale. SD = standard deviation.

	Women	Men	Total	p value for the sex difference
Participants, n (%)	20 (35.7)	36 (64.3)	56 (100)	
Age in years, mean (SD) (range)	36.4 (15.1) (18.0 to 59.6)	28.3 (9.6) (18.5 to 65.3)	31.2 (12.3) (18.0 to 65.3)	**0.041**
Daily smoking, n (%)	12 (66.7)	22 (68.6)	34 (60.7)	1.000
BMI in kg/m^2^, mean (SD)	25.3 (4.4)	24.1 (9.7)	24.5 (6.7)	0.605
Prolactin in mIU/L, mean (SD)	268.5 (141.9)	216.5 (127.4)	235.1 (133.8)	0.178
Use of NSAIDs/antibiotics, n (%)				
Any at baseline	2 (10.0)	0 (0)	2 (3.6)	0.123
NSAIDs	0 (0)	0 (0)	0 (0)	1.000
Antibiotics	2 (10.0)	0 (0)	2 (3.6)	0.123
Any for the entire study period	4 (20.0)	2 (5.6)	6 (10.7)	0.172
NSAIDs	1 (5.0)	2 (5.6)	3 (5.4)	1.000
Antibiotics	2 (10.0)	0 (0)	2 (3.6)	0.123
Alcohol abuse/dependency, n (%)	1 (5.0)	2 (6.3)	3 (5.8)	1.000
Drugs abuse/dependency, n (%)	1 (5.0)	6 (18.2)	7 (13.2)	0.233
Use of Z-hypnotics (throughout whole study period), n (%)	4 (20.0)	9 (25.0)	13 (23.2)	0.925
Use of benzodiazepines (throughout whole study period), n (%)	10 (50.0)	7 (19.4)	17 (30.4)	0.038
Inflammatory disorders, n (%)				
Any	4 (22.2)	6 (15.1)	10 (18.9)	0.719
More than one	0 (0)	1 (2.9)	1 (1.8)	0.524
Rheumatoid arthritis	1 (5.6)	0 (0)	1 (1.8)	0.340
Asthma	1 (5.6)	1 (2.9)	2 (3.6)	1.000
Crohn's disease	0 (0)	1 (2.9)	1 (1.8)	1.000
Psoriasis	0 (0)	1 (2.9)	1 (1.8)	1.000
Irritable bowel syndrome	0 (0)	2 (5.7)	2 (3.6)	0.543
Hyperthyroidism	1 (5.6)	0 (0)	1 (1.9)	0.340
Hypothyroidism	0 (0)	2 (5.7)	2 (3.8)	0.543
ICD-10 diagnosis, n (%)				
Schizophrenia	11 (55.0)	20 (55.6)	31 (55.4)	1.000
Schizotypal disorder	0 (0)	2 (5.6)	2 (3.6)	0.532
Delusional disorder	4 (20.0)	7 (19.4)	11 (19.6)	1.000
Acute and transient psychotic disorders	2 (10.0)	2 (5.6)	4 (7.1)	0.611
Schizoaffective disorder	2 (10.0)	0 (0)	2 (3.6)	0.123
Other nonorganic psychotic disorders	1 (5.0)	0 (0)	1 (1.8)	0.357
Unspecified nonorganic psychotic disorder	0 (0)	5 (13.9)	5 (8.9)	0.148
Duration of illness in years, mean (SD)∗	6.0 (10.0)	3.1 (4.8)	4.0 (12.8)	0.330
PANSS total score at baseline, mean (SD)	76.8 (12.4)	76.0 (13.2)	76.3 (0.7)	0.836
CGI score at baseline, mean (SD)	4.8 (0.8)	4.9 (0.6)	4.9 (0.7)	0.316

### 3.2 Baseline cytokine values

Results are presented in the log-transformed data. At baseline, there were significantly lower levels of IL-4 (diff. −0.98; SD 0.48; p = 0.048) and IL-2 (diff. 1.61; SD 0.381; p = 0.03) in women compared to men (Table 2 and Supplementary Table 1).

**Table 2:** **Changes in cytokine levels from baseline in men and women, and sex differences** The numbers in the table are estimates from a linear mixed-effects model. In this model, baseline cytokine values and the change in cytokine values (log-transformed data) from baseline to the different times are estimated for men and women. The estimates are presented on the log scale. The standard deviations are presented in parentheses, while the p values are presented in square brackets. Statistically significant changes from baseline are presented in bold. The “Difference” row for each cytokine represents a comparison of the change in the cytokine levels from baseline in men versus the change in the cytokine levels from baseline in women at each time. IL = interleukin. TNF-α = tumour necrosis factor α.

	Baseline	1 week	3 weeks	6 weeks	12 weeks	26 weeks	39 weeks	52 weeks
**IL-4**								
**Men**	2 (0.27)	0.02 (0.08) [p = 0.767]	0.06 (0.08) [p = 0.478]	−0.01 (0.09) [p = 0.883]	−0.06 (0.1) [p = 0.573]	−0.09 (0.11) [p = 0.4]	−0.07 (0.12) [p = 0.539]	**−0.46(0.12)[p < 0.001]**
**Women**	1.02 (0.39)	−0.02 (0.11) [p = 0.854]	0.05 (0.11) [p = 0.632]	**0.35 (0.13)[p = 0.006]**	0.3 (0.16) [p = 0.066]	**0.7 (0.16)[p < 0.001]**	**0.63 (0.2)[p = 0.002]**	**0.71 (0.2)[p = 0.001]**
**Difference**	**−0.98(0.48)[p = 0.048]**	−0.04 (0.13) [p = 0.748]	0 (0.14) [p = 0.976]	**0.36 (0.16)[p = 0.022]**	0.36 (0.19) [p = 0.063]	**0.79 (0.2)[p < 0.001]**	**0.7 (0.23)[p = 0.003]**	**1.16 (0.24)[p < 0.001]**

**IL-6**								
**Men**	0.06 (0.22)	0.02 (0.13) [p = 0.901]	−0.13 (0.13) [p = 0.335]	**−0.31(0.15)[p = 0.046]**	−0.01 (0.16) [p = 0.97]	−0.06 (0.18) [p = 0.734]	0.18 (0.19) [p = 0.352]	−0.18 (0.2) [p = 0.37]
**Women**	−0.61 (0.32)	0.06 (0.18) [p = 0.756]	0.33 (0.18) [p = 0.076]	0.32 (0.21) [p = 0.122]	**0.82 (0.27)[p = 0.003]**	**0.74 (0.27)[p = 0.007]**	0.27 (0.34) [p = 0.43]	−0.01 (0.34) [p = 0.969]
**Difference**	−0.67 (0.39) [p = 0.095]	0.04 (0.22) [p = 0.856]	**0.46 (0.23)[p = 0.046]**	**0.63 (0.26)[p = 0.015]**	**0.83 (0.32)[p = 0.01]**	**0.8 (0.33)[p = 0.015]**	0.09 (0.39) [p = 0.823]	0.17 (0.39) [p = 0.671]

**TNF-α**								
**Men**	2.39 (0.32)	0.08 (0.08) [p = 0.301]	0.07 (0.08) [p = 0.354]	0.03 (0.09) [p = 0.726]	−0.06 (0.1) [p = 0.51]	0 (0.11) [p = 0.997]	−0.02 (0.11) [p = 0.845]	**−0.34(0.12)[p = 0.004]**
**Women**	1.6 (0.46)	−0.14 (0.11) [p = 0.202]	0.14 (0.11) [p = 0.204]	0.15 (0.12) [p = 0.233]	0.11 (0.16) [p = 0.502]	**0.43 (0.16)[p = 0.008]**	**0.5 (0.2)[p = 0.012]**	**0.27 (0.2)[p = 0.174]**
**Difference**	−0.8 (0.57) [p = 0.169]	−0.21 (0.13) [p = 0.102]	0.06 (0.13) [p = 0.629]	0.11 (0.15) [p = 0.451]	0.17 (0.19) [p = 0.36]	**0.43 (0.19)[p = 0.026]**	**0.52 (0.23)[p = 0.023]**	**0.61 (0.23)[p = 0.009]**

### 3.3 Longitudinal patterns of cytokine levels in men and women

**Fig. 1:**
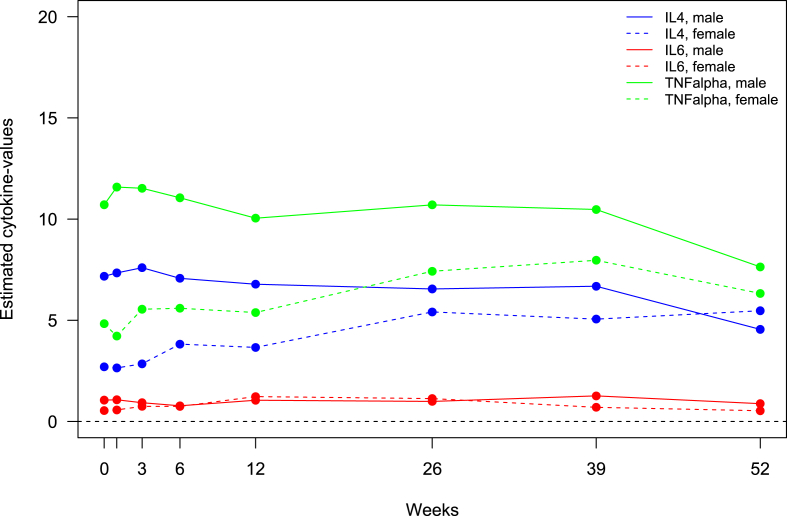
**Cytokine trajectories** The mean changes in cytokine levels (pg/mL) in men (solid lines) and women (dashed lines) during treatment with antipsychotics, compared with baseline measurements before the start of treatment.

We analysed the data for sex-specific effects on the longitudinal patterns of change for each parameter (Fig. 1). Results are presented in the log-transformed data. In men, there were no significant changes in cytokines levels for the first 26 weeks except for a transitory decrease in IL-6 levels at week 6 (−0.31; SD 0.15; p = 0.046) (Table 2). However, at week 52, the IL-4 and TNF-α levels showed a significant reduction compared with baseline (−0.46; SD 0.12; p < 0.001 for IL-4 and -0.34; SD 0.12; p = 0.004 for TNF-α). Similarly, the levels of the combined standardised pro-inflammatory cytokines (Table 3) showed a significant reduction at week 52 (−0.13; SD 0.17; p = 0.005). In women, the IL-4 levels were significantly increased compared with baseline at weeks 6 (0.35; SD 0.13; p = 0.006), 26 (0.7; SD 0.16; p < 0.001), 39 (0.63; SD 0.2; p = 0.002), and 52 (0.71; SD 0.2; p = 0.001). There was a similar increase in IL-6 (week 12: 0.82; SD 0.27; p = 0.003 and week 26: 0.74; SD 0.27; p = 0.007) and TNF-α levels (week 26: 0.43; SD 0.16; p = 0.008 and week 39: 0.5; SD 0.2; p = 0.012), although these changes were not sustained at week 52. For the other cytokines, there were no stable changes in the levels.

**Table 3:** T**he results for the combined standardised pro-inflammatory variable** The combined standardised pro-inflammatory variable comprises the standardised means of IL-1β, IL-6, and TNF-α levels. The numbers in the tables are estimates from a linear mixed-effects model. In this model, baseline cytokine levels (log-transformed data) and the change in cytokine levels from baseline to the different times are estimated for men and women. The estimates are given on the log scale. The standard deviations are presented in the parentheses, while the p values are presented in square brackets. Statistically significant changes from baseline are presented in bold. The p values for women indicate whether the change from baseline is statistically different from males. The “Difference” row represents a comparison of the change from baseline in men versus the change from baseline in women at each time.

	Baseline	1 week	3 weeks	6 weeks	12 weeks	26 weeks	39 weeks	52 weeks
**Men**	0.13 (0.15)	0.07 (0.16) [p = 0.264]	0.06 (0.16) [p = 0.233]	0.09 (0.16) [p = 0.528]	0.03 (0.16) [p = 0.156]	0.1 (0.16) [p = 0.69]	0.04 (0.17) [p = 0.295]	−0.13 (0.17) **[p = 0.005]**
**Women**	−0.11 (0.29)	−0.33 (0.29) [p = 0.064]	−0.19 (0.3) [p = 0.509]	−0.19 (0.3) [p = 0.523]	0.03 (0.32) [p = 0.391]	0.21 (0.32) [p = 0.051]	0.16 (0.33) [p = 0.164]	−0.05 (0.33) [p = 0.747]
**Difference**	0.25 (0.33) [p = 0.457]	0.39 (0.33) [p = 0.242]	0.25 (p = 0.33) [0.458]	0.28 (p = 0.34) [0.413]	0 (0.36) [p = 0.994]	−0.11 (0.36) [p = 0.759]	−0.12 (0.37) [p = 0.756]	−0.08 (0.37) [p = 0.828]

**Table 4:** **Number of participants with data available for analysis at the different time points** This table illustrates number of male and female participants from baseline to each week of the study (attrition was due to several reasons-some patients stopped taking their antipsychotic medication and their subsequent blood samples were not included in the analysis, some dropped out because they moved, some participants did not wish to participate any more, one female patient became pregnant, and we lost contact with some of the patients).

	Baseline	Week 1	Week 3	Week 6	Week 12	Week 26	Week 39	Week 52
Men	36	31	26	18	17	13	11	9
Women	20	17	15	10	5	5	3	3

### 3.4 Sex differences regarding cytokine levels and changes in the levels

**Fig. 2:**
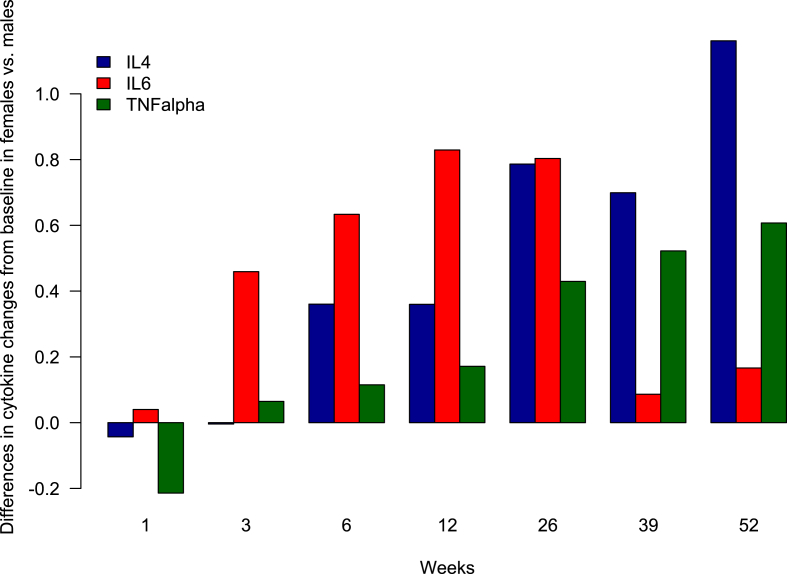
**Longitudinal sex differences in IL-4, IL-6, and TNF-α** This figure illustrates the magnitude of differences in cytokine changes from baseline between women and men. The height of the columns corresponds to the difference between the sexes. Positive columns indicate positive changes in cytokines in women compared with men, and vice versa. The sex difference for IL-4 and TNF-α increased over time during treatment with antipsychotics. For IL-6, the sex difference was more temporary and smaller.

There were significant differences between men and women in how cytokine levels changed from baseline during treatment with antipsychotics (Fig. 2). Results are presented in the log-transformed data. For IL-4, the sex difference was significant at baseline, as reported above, and then at weeks 6 (diff. 0.36; SD 0.16; p = 0.022), 26 (diff. 0.79; SD 0.2; p < 0.001), 39 (diff. 0.7; SD 0.23; p = 0.003), and 52 (diff. 1.16; SD 0.24; p < 0.001). The IL-6 levels changed from baseline differently between the sexes at weeks 3 (diff. 0.46; SD 0.23; p = 0.046), 6 (diff. 0.63; SD 0.26; p = 0.015), 12 (diff. 0.83; SD 0.32; p = 0.01), and 26 (diff. 0.8; SD 0.33; p = 0.015). There was a significant sex difference for TNF-α at weeks 26 (diff. 0.43; SD 0.19; p = 0.026), 39 (diff. 0.52; SD 0.23; p = 0.023), and 52 (diff. 0.61; SD 0.23; p = 0.009). Finally, at week 6 there was a significant sex difference in change of IL-6 level from baseline although not significant when measured for men and women separately (diff. 0.46; 0.23; p = 0.046).

Adjustments for use of benzodiazepines, which was more prevalent among females, did not affect the results – neither longitudinal pattern of cytokine levels in each sex separately, or sex differences in cytokine trajectories.

### 3.5 CRP levels

There were no significant differences in CRP levels between sexes at baseline or during 52 weeks of treatment with antipsychotics. CRP levels did not change during the study period when analysed for each sex separately either, with the exception from week 39, where male patients had a transient increase (2.24; SD 0.71; p = 0.004). See Supplementary Table 2.

## 4 Discussion

The main findings in our study were significant sex-differences in levels of cytokines at baseline and during treatment with olanzapine, aripiprazole, and amisulpride over 12 months in patients previously naïve to antipsychotic drugs. At baseline, female patients had significantly lower levels of IL-4 compared with male patients. During the treatment period, there were sex differences regarding the changes in IL-4, IL-6, and TNF-α levels. Cytokine changes were mainly seen in women. The IL-4 and TNF-α levels in women increased steadily over the study period, whereas the IL-6 levels showed a transient increase until week 26, and then started to decrease.

To the best of our knowledge, this is the first study to prospectively examine sex-specific changes in cytokine levels during treatment with antipsychotics, aside from the shorter study of Ramsey (2013) that analysed the sex-specific effects of 6 weeks of treatment with antipsychotics in 33 antipsychotic-naïve patients with FEP. There was no sex difference for TNF-α, whereas the authors did not measure IL-4 and IL-6.

A recent publication from Ermakov et al. (2023) reported on sex differences in the cytokine levels of patients with schizophrenia. In this cross-sectional case-control study, women were in a more pronounced pro-inflammatory state than men when compared with healthy controls. The levels of several cytokines were higher in women, including IL-4 and IL-6, whereas TNF-α did not differ. When comparing between female and male patients with schizophrenia, there were significantly higher TNF-α levels in male patients. Interestingly, this sex difference was also present for healthy controls. All patients were in the acute phase of schizophrenia and treated with antipsychotics, but due to the cross-sectional design, the authors did not report a longitudinal comparison of untreated individuals versus those who used antipsychotics.

Changes in cytokine levels in patients with schizophrenia, not stratified by sex, have been studied previously. A meta-analysis by Romeo and colleagues in 2018 (Romeo et al., 2018) included 47 studies and analysed changes in cytokine levels in patients with schizophrenia after treatment with antipsychotics. For the FEP group (8 studies), they found reduction in IL-1β (4 studies, p = 000.7), IL-6 (5 studies, p = 0.004) and IL-4 (3 studies p = 0.003). Seven of the 8 studies excluded patients with somatic illnesses or medical treatments that could influence the immune system as well as comorbidity with alcohol or substance abuse/dependency, and treatment duration was from 4 weeks (most of the studies) to 7 months.

In the present study, when we analysed cytokine changes in each sex separately, we found that in accordance with the referred meta-analysis by Romeo et al., a tendency in male patients towards reduction of IL-1β starting at week 12, but without reaching level of statistical significance. Levels of IL-4 in the male group also started to decrease at week 6, and this reduction reached significance at week 52. IL-6 had only a transient reduction at week 26. However, for the female patients of our study IL-4, IL-6, and TNF-α levels increased at several times during treatment with antipsychotics, especially IL-4.

No sex-differences in the levels of CRP at baseline or during the treatment phase were found in our analysis of the drug-naïve participants, while an earlier publication from our group analysing the full sample (both drug-naïve and not, n = 144) found a stronger decline of CRP in men than in women (Fathian et al., 2022).

Our finding of the altered effects on cytokine levels in women treated with antipsychotics could be linked to negative somatic outcomes of schizophrenia such as cardiovascular disease, as inflammation is involved in the development of atherosclerosis (Geovanini and Libby, 2018). Women treated with antipsychotics have a 1.7 times higher risk of developing metabolic syndrome than men and are therefore more prone to develop cardiovascular disease (Brand et al., 2021b).

The mechanism behind an altered inflammatory state in women receiving antipsychotics is not clear. One hypothesis is linked to prolactin (PRL), a peptide hormone mainly secreted in the anterior pituitary gland, with tonic inhibition by dopamine (Costanza et al., 2015). PRL and an elevated pro-inflammatory status in women receiving antipsychotics can be linked in two ways. First, PRL acts as a cytokine and activates both humoral and cellular immunity (Orbach and Shoenfeld, 2007). Second, many antipsychotics are known to reduce oestrogen levels, leading to a disturbance of the menstrual cycle and even diminished menstruation, which is mainly mediated through hyperprolactinemia (Joffe and Hayes, 2008). In the BeSt InTro study, women in the amisulpride and olanzapine groups reported decreased menstruation and had a more pronounced PRL increase during treatment with antipsychotics (Hoekstra et al., 2021). A second theory concerns oestrogen, which at high levels may have a broad anti-inflammatory effect, and specifically reducing the levels of IL-6 and TNF-α (Millas and Duarte, 2021). Therefore, we could hypothesise that the altered inflammatory status in the women in this study is secondary to reduced oestrogen levels, in addition to increased PRL levels. However, we did not analyse oestrogen levels in our patients. Another possible explanation is that premenopausal women in general display more enhanced cellular and humoral immune responses (Klein and Flanagan, 2016).

A steady rise in the levels of mainly IL-4 in women could be a compensatory immunosuppressive mechanism as IL-4 suppresses both IL-6 and TNF-α (te Velde et al., 1990). IL-4 has also been shown to increase insulin sensitivity and glucose tolerance and inhibit adipogenesis (Chang et al., 2012). Secondary analyses of the BeSt InTro study showed that men taking two of the three studied antipsychotics (olanzapine and aripiprazole) had a greater BMI increase compared with women. In addition, men using olanzapine had significantly higher glucose levels throughout the study than women (Hoekstra et al., 2021).

## 5 Limitations and strengths

Our study has several limitations. First, our sample size is small, and we had a significant drop-out rate, especially among women. A fraction of the samples had measurements close to or below the LLOQ. We did not perform a power analysis for these secondary analyses from the BeSt InTro study. We did not measure oestrogen levels. The three antipsychotics that our patients received differ in their pharmacological properties, and we cannot exclude that our findings in differential changes in cytokine levels can be confounded or even explained by that. We do not have data for possible other psychosocial interventions our participants might have been exposed to, prior to this study, that could have influenced their cytokine levels. Finally, we did not correct for multiple comparisons.

The main strength of our study is that it is the first to examine sex differences in cytokine levels during antipsychotic drug treatment in a longitudinal setting. In addition, all patients were antipsychotic naïve, and we only included measures from times when patients used antipsychotics.

## 6 Conclusions

Our analyses demonstrated a stronger immune system response in women than in men during antipsychotic treatment. Cytokine levels changed mainly in women with IL-4 and TNF-α increasing quite steadily during the 1-year follow-up, while IL-6 had a transient increase until week 26. In men, the cytokine levels remained mostly unchanged. The sex differences regarding the changes in the IL-4 and TNF-α levels increased over time.

The mechanisms underlying this difference are not clear, and the clinical implications remain to be uncovered. This study is observational with a hypothesis-generating potential for additional studies. The findings from this study support emerging evidence on sex differences in schizophrenia spectrum disorders and highlight the need for further research to disentangle its significance for the development and treatment of these disorders.

## CRediT authorship contribution statement

**I. Ratke:** Writing – review & editing, Writing – original draft, Investigation, Formal analysis. **A. Torsvik:** Writing – review & editing, Methodology, Formal analysis. **C.A. Bartz-Johannessen:** Writing – review & editing, Visualization, Software, Formal analysis. **F. Fathian:** Data curation, Formal analysis, Writing – review & editing. **I. Joa:** Writing – review & editing, Conceptualization. **S.M. Klæbo Reitan:** Writing – review & editing, Data curation. **E.M. Løberg:** Writing – review & editing, Conceptualization. **M. Rettenbacher:** Writing – review & editing, Data curation, Conceptualization. **S. Skrede:** Writing – review & editing, Formal analysis. **V.M. Steen:** Writing – review & editing, Conceptualization. **E. Johnsen:** Writing – review & editing, Validation, Supervision, Resources, Project administration, Methodology, Investigation, Funding acquisition, Data curation, Conceptualization. **R.A. Kroken:** Writing – review & editing, Validation, Supervision, Resources, Project administration, Methodology, Investigation, Conceptualization.

## Data sharing statement

The data collected are not publicly available due to a lack of approval from the regulatory authorities in Norway.

## Declaration of competing interest

The authors declare the following financial interests/personal relationships which may be considered as potential competing interests: Erik Johnsen reports financial support was provided by 10.13039/501100004787The Research Council of Norway and The 10.13039/100007159Western Norway Regional Health Trust. If there are other authors, they declare that they have no known competing financial interests or personal relationships that could have appeared to influence the work reported in this paper.

## Data Availability

The data that has been used is confidential.
