# Supplementary material for: Sex differences in the peripheral levels of cytokines during 12-month antipsychotic treatment in a drug-naïve schizophrenia spectrum cohort
Source: Brain Behav Immun Health. 2025 Feb 3;44:100959. doi: 10.1016/j.bbih.2025.100959 (PMC11846924; doi:10.1016/j.bbih.2025.100959)
Supplement: Multimedia component 1 [file mmc1.docx]

|  | **Baseline** | **1 week** | **3 weeks** | **6 weeks** | **12 weeks** | **26 weeks** | **39 weeks** | **52 weeks** |
| --- | --- | --- | --- | --- | --- | --- | --- | --- |
| **IFN-γ** |  |  |  |  |  |  |  |  |
| **Men** | 0.49 (0**.**26) | 0.01 (0.265) [p=0.955] | -0.33 (0.27) [p=0.066] | -0.36 (0.287) [p=0.083] | -0.33 (0.298) [p=0.131] | -0.19 (0.312) [p=0.417] | -0.4 (0.325) [p=0.121] | -1.01 (0.344) [p<0.001] |
| **Women** | -0.27 (0.373), [p=0.107] | -0.08 (0.377) [p=0.772] | 0.03 (0.381) [p=0.232] | 0.07 (0.401) [p=0.214] | 0.22 (0.465) [p=0.194] | 0.52 (0.464) [p=0.103] | 0.28 (0.533) [p=0.192] | 0.19 (0.533) [p=0.024] |
|  |  |  |  |  |  |  |  |  |
| **IL1-β** |  |  |  |  |  |  |  |  |
| **Men** | -1.24 (0.3) | -0.19 (0.306) [p=0.384] | -0.13 (0.311) [p=0.55] | 0.05 (0.335) [p=0.835] | -0.16 (0.349) [p=0.548] | -0.29 (0.371) [p=0.329] | -0.35 (0.379) [p=0.26] | -0.58 (0.39) [p=0.075] |
| **Women** | -2.41 (0.6), [p=0.096] | -0.28 (0.615) [p=0.838] | -0.41 (0.629) [p=0.573] | -0.26 (0.614) [p=0.528] | -0.24 (0.733) [p=0.906] | 0.85 (0.73) [p=0.079] | 0.82 (0.812) [p=0.117] | 0.72 (1.009) [p=0.178] |
|  |  |  |  |  |  |  |  |  |
| **IL-10** |  |  |  |  |  |  |  |  |
| **Men** | -1.53 (0.308) | 0.38 (0.314) [p=0.064] | 0.73 (0.32) [p=0.001] | 0.18 (0.345) [p=0.465] | 0.16 (0.353) [p=0.543] | 0.35 (0.371) [p=0.218] | 0.32 (0.387) [p=0.298] | -0.4 (0.397) [p=0.214] |
| **Women** | -1.54 (0.443), [p=0.992] | 0.02 (0.447) [p=0.302] | 0.09 (0.452) [p=0.077] | 0.21 (0.476) [p=0.951] | 0.25 (0.553) [p=0.863] | 0.76 (0.552) [p=0.431] | -0.05 (0.635) [p=0.553] | -1.5 (0.635) [p=0.078] |
|  |  |  |  |  |  |  |  |  |
| **IL-12p70** |  |  |  |  |  |  |  |  |
| **Men** | -0.1 (0.275) | 0.08 (0.277) [p=0.438] | 0.02 (0.278) [p=0.884] | 0.09 (0.284) [p=0.444] | -0.08 (0.288) [p=0.555] | -0.11 (0.291) [p=0.414] | -0.09 (0.296) [p=0.555] | -0.28 (0.299) [p=0.067] |
| **Women** | -0.67 (0.549), [p=0.367] | -0.05 (0.552) [p=0.546] | -0.43 (0.556) [p=0.054] | 0.19 (0.552) [p=0.655] | 0.17 (0.583) [p=0.414] | 0.55 (0.582) [p=0.028] | 0.47 (0.605) [p=0.111] | -0.23 (0.605) [p=0.898] |
|  |  |  |  |  |  |  |  |  |
| **IL-17** |  |  |  |  |  |  |  |  |
| **Men** | 2.58 (0.325) | 0.06 (0.326) [p=0.407] | 0.06 (0.327) [p=0.446] | -0.06 (0.33) [p=0.515] | 0.01 (0.331) [p=0.898] | 0.13 (0.333) [p=0.187] | -0.06 (0.335) [p=0.555] | -0.26 (0.337) [p=0.021] |
| **Women** | 1.54 (0.651), [p=0.168] | -0.04 (0.652) [p=0.538] | 0.01 (0.654) [p=0.772] | 0.23 (0.652) [p=0.099] | 0.2 (0.667) [p=0.392] | 0.22 (0.667) [p=0.711] | 0.56 (0.678) [p=0.017] | 0.05 (0.678) [p=0.229] |
|  |  |  |  |  |  |  |  |  |
| **IL-2** |  |  |  |  |  |  |  |  |
| **Men** | 2.66 (0.265) | 0.09 (0.27) [p=0.602] | 0.06 (0.274) [p=0.749] | -0.24 (0.291) [p=0.232] | -0.02 (0.3) [p=0.917] | -0.28 (0.314) [p=0.231] | 0.08 (0.326) [p=0.742] | -0.09 (0.334) [p=0.739] |
| **Women** | 1.61 (0.381), [p=0.03] | -0.01 (0.385) [p=0.726] | -0.28 (0.388) [p=0.254] | 0.23 (0.407) [p=0.166] | 0.28 (0.467) [p=0.466] | 0.44 (0.466) [p=0.089] | -1.02 (0.531) [p=0.029] | 0.35 (0.531) [p=0.394] |

**Supplementary table 1*:* Changes in cytokine levels from baseline in antipsychotic-naïve men and women**

The numbers in the table are estimates from a linear mixed-effects model. In this model, baseline cytokine levels and the change in cytokine levels (log-transformed data) from baseline to the different times are noted. The estimates are given on the log scale. The standard deviations are presented in the parentheses, while the p values are presented in square brackets. Statistically significant changes from baseline are presented in red. The second line for each cytokine (“Women” represents a comparison of the change in the cytokine levels from baseline in men versus the change in the cytokine levels from baseline in women at each time. IFN-γ=interferon γ. IL=interleukin. TNF-α=tumour necrosis factor α.
