# Supplementary material for: Sex differences in the peripheral levels of cytokines during 12-month antipsychotic treatment in a drug-naïve schizophrenia spectrum cohort
Source: Brain Behav Immun Health. 2025 Feb 3;44:100959. doi: 10.1016/j.bbih.2025.100959 (PMC11846924; doi:10.1016/j.bbih.2025.100959)
Supplement: Multimedia component 2 [file mmc2.docx]

| **CRP (mg/L)** | **Baseline** | **1 week** | **3 weeks** | **6 weeks** | **12 weeks** | **26 weeks** | **39 weeks** | **52 weeks** |
| --- | --- | --- | --- | --- | --- | --- | --- | --- |
| **Male** | 2.01 (0.45) | -0.12 (0.54) [p=0.829] | 0.66 (0.54) [p=0.224] | 0.03 (0.62) [p=0.967] | 0.48 (0.67) [p=0.472] | 0.03 (0.71) [p=0.968] | 2.24 (0.76) [p=0.004] | 0.47 (0.79) [p=0.557] |
| **Female** | 2.49 (0.64) | -1.09 (0.77) [p=0.16] | 0.1 (0.79) [p=0.895] | -0.52 (0.88) [p=0.556] | 0.51 (1.09) [p=0.637] | 0 (1.09) [p=1] | -0.39 (1.34) [p=0.772] | -0.12 (1.34) [p=0.928] |
| **Difference** | 0.48 (0.79) [p=0.549] | -0.97 (0.94) [p=0.303] | -0.55 (0.95) [p=0.565] | -0.54 (1.07) [p=0.614] | 0.03 (1.27) [p=0.98] | -0.03 (1.3) [p=0.982] | -2.62 (1.54) [p=0.09] | -0.59 (1.55) [p=0.707] |

**Supplementary table 2*:* Changes in CRP levels from baseline in antipsychotic-naïve men and women, and sex differences**

The numbers in the table are estimates from a linear mixed-effects model. In this model, baseline CRP values and the change in CRP values from baseline to the different times are estimated for men and women. The standard deviations are presented in parentheses, while the p values are presented in square brackets. Statistically significant changes from baseline are presented in red. The “Difference” row represents a comparison of the change in the CRP levels from baseline in men versus the change in the CRP levels from baseline in women at each time. CRP- C-reactive protein in mg/L.
